# Development of the global inflammatory bowel disease visualization of epidemiology studies in the 21^st^ century (GIVES-21)

**DOI:** 10.1186/s12874-023-01944-2

**Published:** 2023-05-25

**Authors:** Joyce W. Y. Mak, Yang Sun, Julajak Limsrivilai, Murdani Abdullah, Jamilya Kaibullayeva, Domingo Balderramo, Beatriz Iade Vergara, Mukesh Sharma Paudel, Rupa Banerjee, Ida Hilmi, Raja Affendi Raja Ali, Shu Chen Wei, Ka Kei Ng, Mansour Altuwaijri, Paul Kelly, Jesus K. Yamamoto-Furusho, Paulo Gustavo Kotze, Vineet Ahuja, Vui Heng Chong, Hang Viet Dao, Yvonne Abbey, Jessica Y. L. Ching, Agnes Ho, Alicia K. W. Chan, Charles N. Bernstein, Richard B. Gearry, Maria Abreu, David T. Rubin, Iris Dotan, Lindsay Hracs, Gilaad G. Kaplan, Siew C. Ng, On behalf of the GIVES-21 Consortium, Yan Tao, Jiamei Rong, Xiaocui Chen, Huixian Song, Chan Zhou, Yanju Mu, Wenjuan Wei, Xinyu Bai, Satimai Aniwan, Taya Kitiyakara, Kamin Harinwan, Chalermrat Bunchorntavakul, Karjpong Techathuvanan, Phuripong Kijdamrongthum, Tanita Suttichaimongkol, Panu Wetwittayakhlang, Marcellus Simadibrata, Ari Fahrial Syam, Deka Larasati, Tjahjadi Robert Tedjasaputra, Arlyando Hezron Saragih, Hendra Koncoro, Aizhan Kanabayeva, Nazira Kongyrbayeva, Assem Kurmangaliyeva, Yelena Laryushina, Toktarova Perizat, Ayupova Venera, Kaliyakparova Aidana, Ignacio Toscani, Luciana Nicoloff, Marie Howe, Xin Hui Khoo, Wong Choon Heng, Harjinder Singh, Andrew Chua, Khong Wai Hong, Meng-Tzu Weng, Wei-Chen Lin, Hsi-Chang Lee, Chun-Chao Chang, Chun-Chi Lin, Puo Hsien Le, Tien-Yu Huang, Cheuk-Kay Sun, Hsing-Jung Yeh, Yu Hon Ho, Bashaar AlIbrahim, Mohammed Alahmari, Phoebe Hodges, Bright Nsokolo, Yaw Awuku, Michael Li, Kam Hon Chan, Yip Wai Man, Thomas Wong Yun-Sze, Alex Fong

**Affiliations:** 1 Department of Medicine and Therapeutics, The Chinese University of Hong Kong, Hong Kong SAR, China; 2 The First Affiliated Hospital of Kunming Medical University, Kunming, People’s Republic of China; 3 Siriraj Hospital, Bangkok, Thailand; 4 University of Indonesia, Depok, Indonesia; 5 Research Institute of Cardiology and Internal Diseases, Asfendiyarov Kazakh National Medical University, Almaty, Kazakhstan; 6 Hospital Privado Centro Médico de Córdoba, Córdoba, Argentina; 7 CASMU, Las Piedras, Uruguay; 8 National Academy of Medical Sciences, Kathmandu, Nepal; 9 Asian Institute of Gastroenterology, Hyderabad, India; 10 University of Malaya, Kuala Lumpur, Malaysia; 11 The National University of Malaysia, Bangi, Malaysia; 12 National Taiwan University Hospital, Taipei, Taiwan; 13 Conde S. Januário Hospital, Macao SAR, China; 14 Division of Gastroenterology, Department of Medicine, College of Medicine, King Saud University, Riyadh, Saudi Arabia; 15 Tropical Gastroenterology & Nutrition Group, University of Zambia School of Medicine, Lusaka, Zambia; 16 Instituto Nacional de Ciencias Médicas Y Nutrición Salvador Zubirá, Mexico City, Mexico; 17 Pontifícia Universidade Católica Do Paraná (PUCPR), Curitiba, Brazil; 18 All India Institute of Medical Sciences, Asian Institute of Gastroenterology, Hyderabad, India; 19 RIPAS Hospital, Bandar Seri Begawan, Brunei Darussalam; 20 Hanoi Medical University, Hanoi, Vietnam; 21 Euracare Hospital, Accra, Ghana; 22 University of Manitoba IBD Clinical and Research Center, Winnipeg, MB Canada; 23 Department of Medicine, University of Otago, Christchurch, New Zealand; 24 Department of Medicine, University of Miami Miller School of Medicine, Florida, USA; 25 Inflammatory Bowel Disease Center, University of Chicago Medicine, Chicago, IL USA; 26 Division of Gastroenterology, Rabin Medical Center, Petah-Tikva, Israel, Affiliated to the Sackler Faculty of Medicine, Tel Aviv University, Tel Aviv, Israel; 27 Division of Gastroenterology and Hepatology, Department of Medicine, University of Calgary, Calgary, AB Canada; 28 Department of Medicine and Therapeutics, State Key Laboratory of Digestive Disease, The Chinese University of Hong Kong, Hong Kong SAR, China

**Keywords:** Incidence, Prevalence, Environmental, Diet

## Abstract

**Background:**

There is a rapid increase in the incidence of inflammatory bowel diseases (IBD) in newly industrialized countries, yet epidemiological data is incomplete. We herein report the methodology adopted to study the incidence of IBD in newly industrialized countries and to evaluate the effect of environmental factors including diet on IBD development.

**Methods:**

Global IBD Visualization of Epidemiology Studies in the 21st Century (GIVES-21) is a population-based cohort of newly diagnosed persons with Crohn’s disease and ulcerative colitis in Asia, Africa, and Latin America to be followed prospectively for 12 months. New cases were ascertained from multiple sources and were entered into a secured online system. Cases were confirmed using standard diagnostic criteria. In addition, endoscopy, pathology and pharmacy records from each local site were searched to ensure completeness of case capture. Validated environmental and dietary questionnaires were used to determine exposure in incident cases prior to diagnosis.

**Results:**

Through November 2022, 106 hospitals from 24 regions (16 Asia; 6 Latin America; 2 Africa) have joined the GIVES-21 Consortium. To date, over 290 incident cases have been reported. All patients have demographic data, clinical disease characteristics, and disease course data including healthcare utilization, medication history and environmental and dietary exposures data collected. We have established a comprehensive platform and infrastructure required to examine disease incidence, risk factors and disease course of IBD in the real-world setting.

**Conclusions:**

The GIVES-21 consortium offers a unique opportunity to investigate the epidemiology of IBD and explores new clinical research questions on the association between environmental and dietary factors and IBD development in newly industrialized countries.

**Supplementary Information:**

The online version contains supplementary material available at 10.1186/s12874-023-01944-2.

## Introduction

Inflammatory bowel diseases (IBD) are chronic illnesses with significant morbidities and impact on patients’ quality of life due to an unpredictable relapsing and remitting course, development of complications, requirement of hospitalisations, surgeries, and use of costly therapies. It is estimated that more than 7 million people in Europe and the United States will be suffering from IBD by 2030 [1], and its prevalence is estimated to be more than 0.3% in North America, Oceania, and many countries in Europe by 2030 [2–4].

In the 21st century, newly industrialized countries in Asia and Latin America are witnessing a rapidly rising incidence mirroring the epidemiology of Crohn’s disease (CD) seen in the Western world in the latter half of the twentieth century [2, 5, 6]. In contrast, countries in the West are experiencing compounding disease prevalence whereby incidence has stabilized but prevalence is forecasted to reach at least 0.5% of the population in the next decade due to IBD incidence rates being in excess of IBD mortality [7, 8]. This rise in the prevalence of IBD will lead to a substantial increase in the burden of health care systems and society. Mitigating the global burden of IBD will require a concerted effort of disease prevention and healthcare innovations that respond to the changing demographics of IBD population across the world [9].

To date, most epidemiological studies have originated from high income countries and countries in the West whereas there are a lack of prospective, population-based, incidence data from developing countries with lower socioeconomic status [10], including Eastern and Southern Asia, the Middle East, Africa and Latin America [2, 5]. It is of paramount importance to understand the epidemiology of IBD in these regions where data remained scanty in order to quantify the magnitude of the problem globally, to appreciate the public health burden of IBD and to allow appropriate allocation of resources. Defining epidemiology and environmental exposure in IBD in developing and newly industrialized countries can also potentially provide researchers with clues to disease aetiology contributing to the rapid increase of IBD in these regions. In this paper, we report the methodology adopted to study the incidence of IBD in newly industrialized countries and developing countries and to evaluate the impact of dietary factors on IBD development.

## Materials and methods

### Study design

Global IBD Visualization of Epidemiology Studies in the 21st Century (GIVES-21) is a global, prospective, population-based inception cohort study involving 106 sites in 24 regions in Asia, Africa, and Latin America. The primary aim of GIVES-21 is to define the incidence of IBD in a rolling 12-month period starting from October 2021. A buffer period of 3 months is included before and after the study period for all sites to catch any delayed diagnosis impacted by the COVID-19 pandemic. The secondary aims are to determine rates of drug utilisation, hospitalisation, surgery and environmental risk factors including dietary patterns and food additive intake prior to IBD development. Disease incidence is defined as the number of new cases of IBD diagnosed within a specified time period divided by the total size of the at-risk population and will be reported as n per 100,000 persons. Each new incident case within the one-year study period will be followed up in the individual centre every six months for one year after recruitment to confirm disease status. The study design of GIVES-21 was similar to that of the Asia–Pacific Crohn’s and Colitis Epidemiology Study (ACCESS) cohort which had previously reported epidemiology of IBD in 13 countries/ regions across Asia and Australia [6, 11]. We have expanded this well-built and robust study infrastructure and platform to other newly-industrialized and developing regions across Asia, Africa and Latin America [2, 6, 12]. We also included countries within the IBD Emerging Nations Consortium (IBD-ENC) across South Asia, Southeast Asia and the Middle East [13]. Both universities and non-universities hospitals, hospitals in the public and private system were included.

### Study outcomes

The primary outcome is the incidence of IBD defined as the number of new cases diagnosed and captured in a rolling 12 month period starting from October 2021. A buffer period of 3 months is included before and after the study period for all sites to catch any delayed diagnosis impacted by the COVID-19 pandemic. Secondary outcomes include medication utilization, rate of surgery and hospitalization in the regions and environmental and dietary risk factors associated with IBD development.

### Study population and follow-up

#### Selection of custodians and catchment areas

A catchment area was defined as a pre-defined city or town with a well-defined boundary geographically within the country that has a stable population. Local census data were used to define the specific population evaluated. Clinical and demographic (including ethnicity) data describing the population within the catchment area were collected. All hospitals (either university, public or private) provided care for at least 70% of the population within the catchment area. A custodian was selected from each catchment area for overseeing the study and ensuring case ascertainment and accuracy of the data captured.

#### ***Case definition***

A preliminary survey had been conducted at selected regions and general practitioners were informed to refer new cases to specialists within the study area. All incident cases who were 17 years or older, met the diagnostic criteria of IBD and had provided informed consent were recruited. The diagnosis of CD or UC was based on a combination of clinical, biochemical, stool, endoscopic, cross-sectional imaging and histological investigations [14]. Cases of IBD were defined using standard international criteria. Cases of enterocolitis with identified specific causes including gastrointestinal infections, gut ischemia, non-steroidal anti-inflammatory drug-induced enterocolitis and radiation colitis were excluded. Clinical notes of all index cases were reviewed by study custodians to ensure an accurate diagnosis of IBD. Only patients who met the diagnostic criteria of IBD and whose diagnosis remained confirmed at 6-month follow-up had been included as incident cases. At study completion, an audit will be performed by external investigator in six randomly selected sites to confirm case diagnosis and ascertainment.

#### ***Case ascertainment and verification***

When a case of IBD was identified, demographic, clinical symptoms, endoscopic, radiological and histologic reports of the patients were retrieved from the custodian and investigators in the study region and the results would be reviewed by gastroenterologists and the custodian in the study region. Follow-up review of case notes was performed at least 3 months after diagnosis and at every 3 months subsequently for complete case ascertainment and to ensure that all inclusion criteria are met. Finally, we searched all pharmacy records for any new prescription of IBD-related medications (5-aminosalicylates acid, corticosteroids, thiopurines, methotrexate, anti-tumour necrosis factor, anti-integrin, anti-IL12/23, S1P receptor modulators, and JAK inhibitors) and reviewed all endoscopic and histologic reports at each hospital for the study period using diagnostic keywords including “inflammatory bowel disease,” “Crohn's disease,” and “ulcerative colitis” to identify additional incident cases.

### Data collection

Demographics data including age, gender, past medical history, date of diagnosis and type of IBD, disease location and behaviour, smoking status, concomitant medications, family history of IBD, rate of surgery and hospitalization, were collected at the baseline visit. The environmental questionnaire (GIVES-EN) (Appendix A), a questionnaire on current food additive intake (Appendix B) and Dietary Screener (Appendix C) were conducted at the baseline visit. During each follow up, disease behaviour and location according to the Montreal classification, disease activity, medications, surgery and hospitalization since last follow-up would be recorded. Any endoscopy which was performed during the study period was also recorded.

### Environmental and dietary factors

To study the role of environmental factors and dietary food additives in IBD development, a nested case–control study was performed. Incident IBD subjects diagnosed between Oct 2021 and Nov 2022 and healthy controls living in predefined, well-described geographical areas were recruited in a 1:2 ratio. Healthy controls were defined as asymptomatic subjects not having any chronic medical illness and not on any long-term medications. They were randomly selected in the streets, supermarkets or department stores within the same residential area of IBD case and matched by age (± 3 years), gender, ethnicity and geographic location with the IBD cases. Family members of IBD cases were not be included as controls as they might share the same environmental and dietary factors with the IBD case. Demographic data including age, gender, smoking status, past medical history, place of residence and ethnicity were collected at recruitment. The same Environmental questionnaire (GIVES-EN), a questionnaire on current food additive intake and Dietary Screener is administered to cases and controls. No subsequent follow-up was required for healthy controls. All collected information were entered into the same web-based database.

### Development of environmental factors and dietary questionnaires

#### GIVES-21 Environmental Questionnaire

The GIVES-21 Environmental Questionnaire (GIVES-EN) had been developed and validated as a self-administered tool to be used regionally and internationally to determine potential environmental risk factors associated with development of IBD (Appendix A). Based on results from a recent metanalysis [15] and the ACCESS study [12], a total of 36 questions were developed to cover six aspects including demographics, medical history, living or working environment, sleep hygiene, stress level and exercise and diet to be included in the questionnaire. A panel of 10 gastroenterologists with extensive experience in treating IBD were invited to evaluate the relevance of each question, the appropriateness of the response format and the representativeness of each aspect as potential IBD risk factor in their locality. Consensus was reached on 14 out of the 36 questions after the panel’s evaluation. For test–retest reliability, fifteen patients with IBD and fifteen healthy controls were asked to complete the questionnaire at two weeks apart. Cohen’s kappa was calculated for dichotomous and categorical responses and Intraclass Correlation Coefficient (ICC) was calculated for ordinal or continuous responses. Twelve questions showed moderate to excellent reliability with Cohen’s kappa ranged from 0.67 to 1 or with ICC ranged from 0.64 to 0.78. Though two questions fell below 0.4 for Cohen’s kappa at 0 (living on a farm with proximity to farm animals beyond age 18) and at 0.51 (use of antibiotics beyond age 18), the panel of gastroenterologists decided to retain these two questions due to its importance to detect any possible regional differences in terms of IBD risk factors.

#### Current Additive Intake Questionnaire

The Current Additive Intake Questionnaire was a purpose-built questionnaire developed by a collaboration between Hong Kong and Australia with expert input from two specialist dietitians to measure and quantify the intake of food additives (Appendix B) [16]. To accommodate the diversity and availability of processed food in various regions that participate in this study, regional custodians and dietitians had reviewed and provided relevant food names that were common among their respective population’s diet. A dietitian from the study team then reviewed the additive content of the proposed items and included those that contain additives of interest based on the same defined food categories.

#### *Dietary *Screener

We had developed a dietary screener questionnaire which contained a total of nine questions assessing dietary intake of each participant in the past seven days (Appendix C). Intraclass correlation coefficient (ICC) estimates and their 95% confident intervals were based on 2-way mixed-effects model to test for internal consistency on the number of serves reported. ICC of 0.87 indicated a very good reliability. The screener adapted the main traditional Greek diet characteristics, known as MDS from de Groot et al., [17] which was also used in the Hong Kong Chinese study conducted by Woo et al. [18] The original 8-item surveys had been further modified. The ratio of monounsaturated fatty acids to saturated fatty acids were widely distributed among various food groups, and typically assessed via weighted-food records or food frequency questionnaire. Since it was difficult to be assessed with a single question, the item had been deleted. Two additional scores were applied to carotenoid-rich and omega-3 rich food consumption based on the Hong Kong Center for Food Safety NRI for vitamin A [19] and the World Health Organization (WHO) recommendation for omega-3. For each item, consumption at or above the specified amount scored one point, otherwise 0, except for ethanol where consumption below the specified amount would be assigned one point, and otherwise 0. A total of 0 to 9 scores were assigned to each respondent with a score of 5 or more to be considered a high score.

#### Translation

All questionnaires were sent to a professional translation service to translate into the local languages of the participating regions. Translated questionnaires had been reviewed by custodians from each region to confirm the accuracy of languages before dissemination. All of the questionnaires had been translated into 14 languages including, Arabic, Chinese (Simplified), Chinese (Traditional), Hindi, Indonesian, Kazakh, Malay, Nepali, Portuguese, Russian, Spanish, Thai, Tibetan and Viet.

### Study governance

The research protocol had been reviewed by the Co-leads of the Globalisation Cluster from International Organization for the Study of Inflammatory Bowel Disease (IOIBD) (CB, ID, MA, DR, PK, GGK and RG) and Co-leads of GIVES-21 consortium (SCN and GGK). A custodian had been selected from each catchment area for overseeing the study and ensuring completeness of case capture and case ascertainment. The custodians arranged meetings for gastroenterologists, paediatricians, and surgeons within their region to inform them about the study design and case recruitment before study initiation. The custodian within each region cross checked the information from general practitioners, gastroenterologists, paediatricians, surgeons, endoscopy, pathology, and pharmacy records to ensure completeness of case capture and verification. Every three months, custodians from each region will meet with the principal investigators to update the recruitment progress and difficulties. The coordinating project team have regular meetings with the custodians to streamline the study conduction and ensure the strict protocol compliance. Moreover, the data were downloaded and monitored regularly to confirm the data accuracy and consistency.

### Dissemination of results

The findings from the GIVES-21 consortium will be disseminated in a variety of ways including abstracts, posters and presentations at conferences and published manuscripts in peer-reviewed journals. Besides, a web-based interactive atlas showing data generated from the GIVES-21 consortium has been created using ArcGIS Online (www.esri.com/en-us/home) for data visualisation. A pilot interactive website for the burden of IBD is provided in the following link: https://ucalgary.maps.arcgis.com/apps/MapSeries/index.html?appid=93e520cd04624e128f7acbb238f7ef87

### Preliminary results

The GIVES-21 consortium represented custodians from 24 regions across Asia, Africa and Latin America. including Argentina, Brazil, Brunei Darussalam, Chile, Colombia, Ghana, Hong Kong SAR of China, India, Indonesia, Kazakhstan, Kunming (China), Macao SAR of China, Malaysia, Mexico, Nepal, Philippines, Saudi Arabia, Singapore, Taiwan, Thailand, United Arab Emirates, Uruguay, Vietnam and Zambia (Table 1). Recruitment started in October 2021 and is currently ongoing. By the end of November 2022, over 290 IBD incident cases had been recorded in the REDCAP online database and 54% were male. More than half of the subjects were diagnosed with UC. The vast majority of subjects had diverse ethnicities. Based on classification by the United Nations Geoscheme Region [20], 39% and 28%, respectively, of the new IBD cases were from Eastern Asia and South-eastern Asia. Around 16% of the new cases were identified from regions from Central Asia and Southern Asia. Around 5% of the new IBD cases were reported to be from each of Eastern Africa, Central America, South America and Western Asia. The second stage of capturing additional incident cases by reviewing case notes, pharmacy records, endoscopy and histology records is in progress. Nearly 90% of the subjects had defined their disease phenotype based on Montreal classification.

**Table 1:** Preliminary progress of GIVES-21 by regions

Regions included in GIVES-21 (as of September 2022)		
1. Argentina 2. Brazil 3. Brunei Darussalam 4. Chile 5. Colombia 6. Ghana 7. Hong Kong SAR 8. India 9. Indonesia 10. Kazakhstan	11. Kunming, China 12. Macao SAR 13. Malaysia 14. Mexico 15. Nepal 16. Philippines 17. Saudi Arabia 18. Singapore 19. Taiwan	20. Thailand 21. United Arab Emirates 22. Uruguay 23. Vietnam 24. Zambia

The second aim of GIVES-21 was to develop a research platform for defining environmental exposure including dietary factors. Each incident case had provided us with data on their environmental exposure and food additive intake prior to diagnosis. The GIVES-EN questionnaire aimed to incorporate different aspects of the environmental risk factors for IBD in different parts of the world whereby lifestyle and globally may differ. The questionnaire comprised 14 questions and covered socioeconomic status and living conditions, early life exposure such as weaning, pet exposure and antibiotic use, smoking history, exercise and stress level. To understand exposure to food additives, we had collected data on food habits in the last year prior to diagnosis especially of non-homemade (processed) products. Up until November 2022, over 95% of enrolled IBD patients (cases) and over 98% of enrolled healthy subjects (controls) had completed the GIVES-EN questionnaire, Dietary Screener and Current Additive Intake Questionnaire at baseline.

## Discussion

To the best of our knowledge, this is one of the most diverse and comprehensive epidemiology platform covering more than 20 countries in Asia, Latin America and Africa studying the epidemiology of IBD and environmental and dietary factors associated with development of IBD with a focus in newly industrialized and developing regions. The success of this initiative relied on the development of a global network of gastroenterologists, surgeons and primary care physicians who have clinical and/or epidemiological interest in IBD. This global network served as custodians to ensure the accuracy of the data presented on our online atlas. Custodians from regions that currently lacked population-based data had taken on leading role in conducting ground-level epidemiological research to establish the burden of IBD in their respective countries within GIVES-21. The unique outcome of the GIVES-21 consortium would include an updated incidence of IBD in knowledge-gap areas, understanding the geographical effect on disease activity, differences in disease phenotype and understanding the association of food additives with the development of IBD in newly industrialized regions.

An ideal epidemiologic incidence study should be population-based, with uniform diagnostic criteria and strict case definitions, complete case ascertainment over a defined period time based on a stable and well-defined population. The low population incidence of IBD in many low- and middle-income countries made this challenging so we adopted an approach to include custodians who were capable to engage IBD specialists, pathologists, radiologists, endoscopists and colorectal surgeons within sentinel hospitals that diagnosed and followed-up IBD patients, using a standardized criteria and infrastructure. We recognize that any missed new IBD incident cases would contribute to under representation of incidence data. To overcome missing incident cases, initiation meetings were organized for all custodians and their respective networks of investigators including surgeons, gastroenterologists, pathologists and IBD nurses across public and private sectors to raise awareness and enhance the comprehensiveness of the enrolment of IBD incident cases. This also involved identifying patients that had yet to receive a formal IBD diagnosis (ie. Indeterminate cases or suspected cases) but had been treated with milder form of IBD medications during the study period. Medical records were screened by the custodians and investigators to exclude cases whose final diagnosis were of infectious causes. We anticipated that smaller and more stable catchment areas that were geographically contained with limited access to external IBD care would achieve close to 100% capture of all incident IBD cases. Most of the regions had a well-defined catchment area and an established network of IBD healthcare providers that included public secondary and tertiary hospitals with close network with primary care and would be able to capture at least 90% of the incident cases during the study period.

IBD is believed to be related to the activation of the intestinal mucosal immune system in response to dysbiosis of the gastrointestinal tract in genetically susceptible individuals [21, 22]. Various environmental risk factors have been implicated in the pathogenesis of IBD, one of which is diet [23]. Certain nutrients and food additives can impact on the gut microbiome and their interactions with the host may play a role in the pathogenesis of IBD [24, 25]. Consumption of ultra-processed food had increased over the last decade in industrialized countries, and epidemiological studies had found associations between ultra-processed food consumption and chronic diseases. Emerging preclinical evidence show that consumption of food additives was associated with gut dysbiosis and may contribute to the development of gut inflammation [26]. Two commonly used emulsifiers, carboxymethylcellulose and polysorbate-80 (P-80) in low concentration have been shown to induce low grade inflammation in gut and colitis in predisposed mice via alterations in the gut microbiome [27, 28], Further studies are now required to identify the potential culprit in ultra-processed food, such as a poor nutritional composition or the presence of food additives. While evidence of varying quality had identified potential harmful or beneficial dietary components, physicians and patients currently do not have guidance as to which foods are safe, may be protective or deleterious for their apart from a recent guidance document compiled by the nutrition cluster of the International Organization for the Study of Inflammatory Bowel Diseases (IOIBD) [29]. We have included the food additive questionnaires to capture these data in a diverse population that hoped to shed insight into diet impact on different IBD populations.

Performing questionnaire study across different regions and populations might have inherent issues due to cultural and dietary differences. To limit this bias, we have invited regional custodians and dietitians to review all questionnaires to accommodate the diversity of environmental and dietary factors and availability of processed food in each respective region. Like all questionnaire studies, missing data and recall bias especially on questions regarding early life factors and dietary intake are inevitable. The inception nature of this study lowers the rate of recall bias when compared with hospital-based or retrospective cohort, although any recall bias is unavoidable in questionnaire-based study. We believe that some factors may be less affected by recall bias, such as breast feeding, current food additives intake. Validation of questionnaires generally require forward and backward translation which was not formally performed in this study. However, to overcome the potential pitfalls without forward and backward translation, we have employed professional translation service to translate them into local languages and each regional custodian were actively involved in reviewing each question to ensure the languages are adaptable and understandable by their respective population before disseminating them to our study participants.

Unlike Nordic countries that have healthcare insurance code and nationwide registry to capture IBD cases, no such systems exist in most areas in the world whereby IBD data are lacking. One of the major challenges in conducting incidence study where by insurance of also include potential difficulties such as identifying all incident IBD cases in the catchment areas, especially in areas where technology or internet coverage is less developed. Regions that had a mobile population or a disjointed healthcare network with a diverse range of IBD care providers may face difficulties to capture all IBD incident cases. We anticipate that a small proportion of centers would only be able to provide hospital-based data on incidence which would lead to an underestimation of new cases. Nonetheless, there is a current knowledge gap on the epidemiology and outcomes of patients with IBD in these newly industrialized regions. Any hospital-based data would still be valuable. Another potential challenges would be the continuation of follow-up for newly recruited patients with IBD, especially during the current COVID-19 pandemic. We anticipated that least 80% of the newly recruited incident IBD cases will complete 6-month follow up and 50% will complete a 12-month follow up by June 2023.

In conclusion, the burden of IBD is rising globally, with substantial variation in levels and trends of disease in different countries and regions. Understanding these geographical differences is crucial for formulating effective strategies for preventing and treating IBD. We believe new data arising from the GIVES-21 consortium will provide essential data to knowledge users, healthcare providers, policy makers who can prepare their healthcare systems for the rising global burden of CD and UC.

## Supplementary Information

**Additional file 1:** **Appendix A.** GIVS-21 Environmental Questionnaire (GIVES-EN). **Appendix B.** GIVES-21 Current Additive Intake. **Appendix C.** Dietary Screener.

## Data Availability

The datasets generated and/or analysed during the current study are not publicly available due as the study is still ongoing but are available from the corresponding author on reasonable request upon completion of study.

## Abbreviations

CD: Crohn’s disease
UC: Ulcerative colitis

## References

[1] Hammer T, Langholz E. The epidemiology of inflammatory bowel disease: balance between East and West? A narrative review. Digestive Medicine Research 2020;3. https://cdn.amegroups.cn/journals/ales/files/journals/34/articles/6855/public/6855-PB4-6289-R2.pdf.

[2] Ng SC, Shi HY, Hamidi N (2017). Worldwide incidence and prevalence of inflammatory bowel disease in the 21st century: a systematic review of population-based studies. Lancet.

[3] Coward S, Clement F, Benchimol EI (2019). Past and Future Burden of Inflammatory Bowel Diseases Based on Modeling of Population-Based Data. Gastroenterology.

[4] Molodecky NA, Soon IS, Rabi DM (2012). Increasing incidence and prevalence of the inflammatory bowel diseases with time, based on systematic review. Gastroenterology.

[5] Kotze PG, Underwood FE, Damiao A (2020). Progression of Inflammatory Bowel Diseases Throughout Latin America and the Caribbean: A Systematic Review. Clin Gastroenterol Hepatol.

[6] Ng SC, Tang W, Ching JY (2013). Incidence and phenotype of inflammatory bowel disease based on results from the Asia-pacific Crohn's and colitis epidemiology study. Gastroenterology.

[7] Kaplan GG, Windsor JW (2021). The four epidemiological stages in the global evolution of inflammatory bowel disease. Nat Rev Gastroenterol Hepatol.

[8] Kaplan GG (2015). The global burden of IBD: from 2015 to 2025. Nat Rev Gastroenterol Hepatol.

[9] Ananthakrishnan AN, Kaplan GG, Ng SC (2020). Changing Global Epidemiology of Inflammatory Bowel Diseases: Sustaining Health Care Delivery Into the 21st Century. Clin Gastroenterol Hepatol.

[10] Collaborators GBDIBD (2020). The global, regional, and national burden of inflammatory bowel disease in 195 countries and territories, 1990–2017: a systematic analysis for the Global Burden of Disease Study 2017. Lancet Gastroenterol Hepatol.

[11] Ng SC, Kaplan GG, Tang W (2019). Population Density and Risk of Inflammatory Bowel Disease: A Prospective Population-Based Study in 13 Countries or Regions in Asia-Pacific. Am J Gastroenterol.

[12] Ng SC, Tang W, Leong RW (2015). Environmental risk factors in inflammatory bowel disease: a population-based case-control study in Asia-Pacific. Gut.

[13] Banerjee R, Pal P, Hilmi I (2022). Emerging IBD demographics, phenotype and treatment in South Asia, South-East Asia and Middle East: Preliminary findings from the IBD-Emerging Nations' Consortium. J Gastroenterol Hepatol.

[14] Maaser C, Sturm A, Vavricka SR (2018). ECCO-ESGAR Guideline for Diagnostic Assessment in IBD Part 1: Initial diagnosis, monitoring of known IBD, detection of complications. J Crohns Colitis.

[15] Piovani D, Danese S, Peyrin-Biroulet L (2019). Environmental Risk Factors for Inflammatory Bowel Diseases: An Umbrella Review of Meta-analyses. Gastroenterology.

[16] Trakman G, Lin WYY, Wilson-O'brien A (2019). P772 Development and validation of tools to assess food additive intake: the ENIGMA study. J Crohns Colitis.

[17] de Groot LC, van Staveren WA, Burema J (1996). Survival beyond age 70 in relation to diet. Nutr Rev.

[18] Woo J, Woo KS, Leung SS (2001). The Mediterranean score of dietary habits in Chinese populations in four different geographical areas. Eur J Clin Nutr.

[19] Lakatos PL (2009). Environmental factors affecting inflammatory bowel disease: have we made progress?. Dig Dis.

[20] United Nations Statistics Division. Standard country or area codes for statistical use (M49). https://unstats.un.org/unsd/methodology/m49/.

[21] Loftus EV (2004). Clinical epidemiology of inflammatory bowel disease: Incidence, prevalence, and environmental influences. Gastroenterology.

[22] Kaplan GG, Ng SC (2017). Understanding and Preventing the Global Increase of Inflammatory Bowel Disease. Gastroenterology.

[23] Ananthakrishnan AN (2015). Epidemiology and risk factors for IBD. Nat Rev Gastroenterol Hepatol.

[24] Maslowski KM, Mackay CR (2011). Diet, gut microbiota and immune responses. Nat Immunol.

[25] Lomer MC, Thompson RP, Powell JJ (2002). Fine and ultrafine particles of the diet: influence on the mucosal immune response and association with Crohn's disease. Proc Nutr Soc.

[26] Laudisi F, Stolfi C, Monteleone G. Impact of Food Additives on Gut Homeostasis. Nutrients. 2019;11:2334. 10.3390/nu11102334. 10.3390/nu11102334 PMC6835893 31581570

[27] Chassaing B, Koren O, Goodrich JK (2015). Dietary emulsifiers impact the mouse gut microbiota promoting colitis and metabolic syndrome. Nature.

[28] Suez J, Korem T, Zilberman-Schapira G (2015). Non-caloric artificial sweeteners and the microbiome: findings and challenges. Gut Microbes.

[29] Levine A, Rhodes JM, Lindsay JO (2020). Dietary Guidance From the International Organization for the Study of Inflammatory Bowel Diseases. Clin Gastroenterol Hepatol.

