# Supplementary material for: Development of the global inflammatory bowel disease visualization of epidemiology studies in the 21st century (GIVES-21)
Source: BMC Med Res Methodol. 2023 May 25;23:129. doi: 10.1186/s12874-023-01944-2 (PMC10209572; doi:10.1186/s12874-023-01944-2)
Supplement: Supplementary file 1 — Additional file 1: Appendix A. GIVS-21 Environmental Questionnaire (GIVES-EN). Appendix B. GIVES-21 Current Additive Intake. Appendix C. Dietary Screener. [file 12874_2023_1944_MOESM1_ESM.pdf]

Appendix A - GIVS-21 Environmental Questionnaire (GIVES-EN)

The objective of this questionnaire is to incorporate different aspects of the environmental risk factors for Inflammatory Bowel Disease globally. The questionnaire comprises 14 questions. This is for cross-sectional use and not for making any diagnosis.

Instruction: All answers in each question are mutually exclusive. Please read each question carefully then tick a box and write down the answer in the space provided if necessary.

1. What is your highest level of education?	☐ $_1$No school		☐ $_2$Primary school
	☐ $_3$Secondary school or College		☐ $_4$University or above
2. Do any of your parents, siblings, spouse or children have IBD? (Please circle the relative who have IBD)	☐ $_1$Yes (Father/ Mother/ Sibling/ Spouse/ Child)		☐ $_2$No
3. How did your mother give birth to you?	☐ $_1$Vaginal	☐ $_2$Caesarean-section	☐ $_3$Not sure
4. Were you breastfed as a baby?	☐ $_1$Yes: _____months	☐ $_2$No	☐ $_3$Not Sure
5. Did you take antibiotics in the following age groups?	0 – 10 years	☐ $_1$Yes	☐ $_2$No
	>10 – 18 years old	☐ $_1$Yes	☐ $_2$No
	>18 years old to current	☐ $_1$Yes	☐ $_2$No
6. Where did you live in the following age groups for more than 50% of the time? (Countryside is defined as a land not in towns, cities, or industrial areas)	0 – 10 years	☐ $_1$City	☐ $_2$Countryside
	>10 – 18 years old	☐ $_1$City	☐ $_2$Countryside
	>18 years old to current	☐ $_1$City	☐ $_2$Countryside
7. Did you keep any pet in your home in the following age groups? (If yes, please write down the pet you keep)	0 – 10 years	☐ $_1$Yes ____________	☐ $_2$No
	>10 – 18 years old	☐ $_1$Yes ____________	☐ $_2$No
	>18 years old to current	☐ $_1$Yes ____________	☐ $_2$No
8. Did you live on farm where you had close proximity with farm animals in the following age groups? (If yes, please write down the farm animal you keep)	0 – 10 years	☐ $_1$Yes ____________	☐ $_2$No
	>10 – 18 years old	☐ $_1$Yes ____________	☐ $_2$No
	>18 years old to current	☐ $_1$Yes ____________	☐ $_2$No
9. Did you smoke in the following age groups? (Use of e-cigarettes/ cigars/ or other tobacco products at least once a month for 6 consecutive months or above)	0 – 10 years	☐ $_1$Yes	☐ $_2$No
	>10 – 18 years old	☐ $_1$Yes	☐ $_2$No
	>18 years old to current	☐ $_1$Yes	☐ $_2$No
10. Did your household member smoke when you were in the following age groups? (Use of e-cigarettes/ cigars/ or other tobacco products at least once a month for 6 consecutive months or above)	0 – 10 years	☐ $_1$Yes	☐ $_2$No
	>10 – 18 years old	☐ $_1$Yes	☐ $_2$No
	>18 years old to current	☐ $_1$Yes	☐ $_2$No

Appendix A - GIVS-21 Environmental Questionnaire (GIVES-EN)

<u>For healthy controls</u>, please answer the following questions (No. 11-14) in regard to your experiences in the past 12 months.

<u>For IBD patients</u>, please answer the following questions (No. 11-14) in regard to your experiences in the past 12 months *before* your IBD diagnosis

11. How often have you felt nervous and “stressed”?	☐ 0 Never<br>☐ 1 Almost never<br>☐ 2 Sometimes<br>☐ 3 Fairly Often<br>☐ 4 Very Often
12. How often have you felt that you were unable to control the important things in life?	☐ 0 Never<br>☐ 1 Almost never<br>☐ 2 Sometimes<br>☐ 3 Fairly Often<br>☐ 4 Very Often
13. How often have you found that you could not cope with all the things that you had to do?	☐ 0 Never<br>☐ 1 Almost never<br>☐ 2 Sometimes<br>☐ 3 Fairly Often<br>☐ 4 Very Often
14. How often do you have physical activities (Walking, jogging, cycling, swimming or similar activities for more than 30minutes)	☐ 0 Never<br>☐ 1 Rarely<br>☐ 2 At least once a month<br>☐ 3 At least once a week<br>☐ 4 At least once a day

Appendix B – Current Additive Intake

Current Additive Intake

We would like to know about your food habits in the last year. Please mark "✓" / "X" (yes/no) if you had each of the listed foods or drinks in the last year. If you select "yes", give an idea of how often you had the food per day, or week, or month, or year in average. Fill out one box only.

Please estimate using **household measures** e.g. 1 Tablespoon, 1 cup or other common description of food sizes e.g. 1 piece, 1 bowl (Please refer to the measurements showing in the "Serving size reference").

Please note, the foods listed below are all referring to ***non-homemade (processed) products***.

	Yes/No had these foods ✓ Or X **(If yes, pls fill in how many times)**	Times per day	Times per week	Times per month	Times per year	Average amount for each time consumed (can be a fraction) Circle the appropriate Unit
		Select one of the above, and fill in with integer (fill in one box only)				
1. Store-bought pasta, pasta salad, or noodles (E.g. packet spaghetti, pre-made ravioli, pre-made pasta salad)						Bowl(s)
2. Store bought bread (E.g. packaged sliced bread, packaged wraps)						Slice(s)
3. Crackers (E.g., rice-cakes, savoury crackers, potato chips)						Piece(s)
4. Packaged soup (E.g. Cup-of Soup, broth in a can , soup mixes by Heinz or Campbell)						Bowl(s)
5. Processed meats and products (E.g. deli meat, sausages, burgers, chicken nuggets)						Piece(s)
6. Processed seafood and products (E.g. canned clams, canned salmon, fish fingers, fried seafood, crab cakes)						Piece(s)
7. Processed vegetables and products (E.g. canned vegetables, pickled vegetables, fermented vegetables, vegetable juice)						Bowl(s)
8. Processed fruits and products (E.g. dried fruit, canned fruit, fruit compote, jam, fruit juice)						Bowl(s)
9. Flavoured milk (E.g. Moo™ chocolate milk, Dare™ iced coffee)						mL
10. Plain sour dairy or cream (E.g. plain yoghurt, sour cream, sour milk, kefir, pouring cream, whipped cream)						mL
11. Milky desserts (E.g., chocolate mousse, vanilla pudding, flavoured yoghurt)						mL /cup

Appendix B – Current Additive Intake

	Yes/No had these foods ✓ Or X **(If yes, pls fill in how many times)**	Times per day	Times per week	Times per month	Times per year	Average amount for each time consumed (can be a fraction) Circle the appropriate Unit
		Select one of the above, and fill in with integer (fill in one box only)				
12. Sugars or syrups (E.g. golden syrup, maple syrup, sugar toppings)						Tbsp(s)
13. Chewing gum (E.g. Extra™)						Piece(s)
14. Sweet baked foods (E.g. cakes, biscuits, muesli bars, gluten-free cake)						Piece(s) / Bar(s)
15. Bottled tea (E.g. Lipton® ice tea, Teavena®)						mL
16. Bottled coffee and coffee substitutes (E.g. instant coffee, espresso coffee, Echo™)						mL
17. Sports Drinks (E.g. Powerade™, Gatorade™)						mL
18. Diet drinks or sugar substitutes (E.g. Diet Coke™, Pepsi-max™, diet iced-tea, drinks sweetened with Equal™, Splenda™, Sweet 'n' Low™)						mL
19. Alcoholic drinks (E.g. beer, wine, cider, spirits)						mL
20. Vitamin pills (E.g. fish oil capsules, multivitamin pills)						Pill(s)
21. Whey proteins and products (E.g. protein bar, protein powder)						Gram(s)/ Tbsp(s)
22. Store bought egg-based desserts (E.g. custard)						Piece(s)
23. Milk powder (E.g. instant dry milk)						Tbsp(s)
24. Salad dressing (E.g. mayonnaise, tartar sauce, Thousand Island dressing)						Tbsp(s)
25. Sweets or lollies (E.g. liquorice, mints, skittles, marshmallows, chocolates)						Piece(s)
26. Coffee-whitener (E.g. Coffee-mate™)						mL
27. Processed juice or flavoured soy milk (E.g. Sesame soymilk)						mL

Name of the interviewee: ________________________________ Signature: _______________________________

**Verification**	
Name of research staff: __________________________	Signature of research staff: __________________________

Appendix C – Dietary Screener

Dietary Screener

Inflammatory Bowel Disease Patient: We are learning your dietary pattern 3 months prior to the onset of any IBD symptoms. Please indicate below your consumption of each food groups in number of serve(s) per day, in a regular week. For example, on average, you have 1 serve of alcohol per week. Please write down $\frac{1}{7}$ serve/day.

Healthy Control: We are learning your dietary pattern during the last 3 months. Please indicate below your consumption of each food groups in number of serve(s) per day, in a regular week. For example, on average, you have 1 serve of alcohol per week. Please write down $\frac{1}{7}$ serve/day.

1. How much alcohol did you drink?<br>**1 serve = 250mL beer or 100mL wine or 30mL hard liquor**	______ Serve/day
2. How much legumes did you eat?<br>**1 serve = 1 tablespoon = 22.5g**	______ Serve/day
3. How much cereals did you eat?<br>**1 serve =~1 bowl of Rice and Noodles = ~200g**	______ Serve/day
4. How much fruits did you eat?<br>**1 serve = 1 medium sized fruit eg. Apple 150g or 2 small sized fruit eg. Kiwi 2x 75g**	______ Serve/day
5. How much vegetables did you eat?<br>**1 serve = 80g = half bowl**	______ Serve/day
6. How much meats or meat products did you eat?<br>**1 serve = 1 ounce of meat = 28g = 1 matchbox**	______ Serve/day
7. How much dairy products did you have?<br>**1 serve = milk 240ml or Yogurt 150g**	______ Serve/day
8. How much red and orange fruits or vegetables did you eat?<br>**1 serve = 80g = half bowl carrots = 1 bowl of tomatoes**	______ Serve/day
9. How much oily fish (herring, mackerel, salmon, or yellow croaker) did you eat?<br>**1 serve = 100g = 1 fish steak or 1 palm size**	______ Serve/day
